# A new sand-dwelling species of *Rineloricaria* (Siluriformes, Loricariidae) from the Eastern Brazilian Basin

**DOI:** 10.3897/zookeys.1269.155896

**Published:** 2026-02-13

**Authors:** Beatriz Rodrigues da Cruz, Jonatas Santos Lima Pereira, Claudio Oliveira, Guilherme José da Costa Silva

**Affiliations:** 1 Pós-Graduação em Saúde Única, Universidade Santo Amaro, São Paulo, São Paulo, Brazil Universidade Estadual Paulista Júlio de Mesquita Filho (UNESP) Botucatu Brazil; 2 Museu de Zoologia da Universidade de São Paulo, Av. Nazaré, 481, Ipiranga, São Paulo, 04263-000, SP, Brazil Museu de Zoologia da Universidade de São Paulo São Paulo Brazil; 3 Instituto de Biociências, Universidade Estadual Paulista Júlio de Mesquita Filho (UNESP), Botucatu, São Paulo, Brazil Universidade Santo Amaro São Paulo Brazil

**Keywords:** Armoured catfishes, Brazilian Crystalline Shield, freshwater fishes, integrative taxonomy, new taxon

## Abstract

We describe *Rineloricaria harenae***sp. nov**., a sand-dwelling armoured catfish from the Itapocú River, Jaraguá do Sul, Santa Catarina, Brazil. The new species is diagnosed by a distinctive combination of characters: (1) four lateral plate series, with the mid-dorsal series absent; (2) abdomen only partially covered by central plates that do not reach the pectoral-fin insertion; (3) plates of the central abdominal complex arranged in an approximately triangular pattern; (4) cleithral (scapular) region entirely naked or bearing a few small, isolated plates; and (5) presence of five dark transverse bars on the dorsal surface of the body. Mitochondrial COI data corroborate species distinctiveness, and phylogenetic inference under the GTR model recovers *R. harenae* as a well-supported lineage. The species is currently known only from the type locality, a shallow (< 1 m), sandy reach showing early siltation, suggesting potential vulnerability. Although it exhibits canonical traits of sand-dwelling groups (four lateral plate series, broad naked snout, filamentous upper caudal ray), it differs from sand-dwelling congeners by displaying an abdominal plating pattern typical of rock-dwelling groups. This raises hypotheses about the origin and functional role of ventral plates in *Rineloricaria* and highlights conservation needs for low-order sandy habitats.

## Introduction

*Rineloricaria* Bleeker, 1862, is the most diverse genus of the subfamily Loricariinae (family Loricariidae) with 75 valid species (Fricke et al. 2025). These catfishes are widely distributed from Central America (Costa Rica) to South America (Argentina) on both sides of the Andes and occupy a wide variety of habitats and drainages (Reis and Cardoso 2001; Covain and Fisch-Muller 2007; Fichberg and Chamon 2008; Vera-Alcaraz et al. 2012). With rare exceptions, species of *Rineloricaria* usually occupy small rivers with moderate to strong currents and depths almost never greater than 1 m (Costa-Silva et al. 2021).

*Rineloricaria* are slender, bottom-dwelling fishes that often exhibit pronounced sexual dimorphism; for example, mature males develop rows of hypertrophied odontodes (dermal bristles) on the sides of the head and dorsal surface of the pectoral fin and in the predorsal area (Isbrücker and Nijssen 1992; Fichberg and Chamon 2008). The Brazilian Crystalline Shield is home to rivers with high environmental diversity and great alternation between lentic and lotic stretches, including the drainages of southern Brazil, which have many *Rineloricaria* species described from the region (Ribeiro 2006; Ghazzi 2008; Rodriguez and Reis 2008; Albert and Reis 2011; Buckup 2011).

Rodriguez and Reis (2008) proposed a functional classification of *Rineloricaria* based on habitat preference and phenotypic traits, in which species of *Rineloricaria* are assembled into two groups: species inhabiting rocky substrates, and species inhabiting sandy substrates. Species from the rock group usually display a dark-brown or dark-grey background colour, five series of lateral plates (mid-dorsal series is present), a broad, rounded snout with an oval, naked tip area that does not reach the first pore of the lateral sensory canal, and the presence of a dorsal-fin spinelet. The abdomen in rock-dwelling species is highly variable, ranging from fully plated to almost completely naked, and variation is both intra- and interspecific. In contrast, the species from the sand group exhibit a light-brown background colouration, four series of lateral plates (the mid-dorsal series is absent), a naked area at on the tip of the snout extending to the first pore of the lateral sensory canal, and the absence of a dorsal-fin spinelet. The abdomem and cleitral region in sand-dwelling species are consistently covered by plates.

Here, we describe a new sand-dwelling species of *Rineloricaria* from the Itapocú River, municipality of Jaraguá do Sul. This species retains nearly all diagnostic traits of the sand group but differs by having its abdomen only partially covered by plates. This uncommon condition results in a unique character combination within the genus, highlighting an intriguing evolutionary scenario and raising questions about the origin and functional significance of abdominal plating.

## Material and methods

Measurements were taken with digital callipers on the left side of the specimens whenever possible. Measurements, abbreviations and delimitations of abdominal plates follow (Vera-Alcaraz et al. 2012). Lateral plates were counted according to the serial homology and terminology proposed by Schaefer (1997). Morphometrics are given as percentages of standard length (**SL**), except for head subunits presented as percentages of head length (**HL**). Pectoral, pelvic, dorsal, and anal-fin rays are represented in Arabic numerals. Specimens were deposited at the Laboratório de Biologia e Genética de Peixes (**LBP**), Botucatu, São Paulo, Brazil and Museu de Zoologia da Universidade de São Paulo (**MZUSP**), Universidade de São Paulo, São Paulo, Brazil.

The phylogenetic position of the new species was inferred using 147 sequences of the cytochrome c oxidase subunit I (COI) gene. Sequences were aligned in MUSCLE (Madeira et al. 2024) using default parameters, and the final alignment was edited in BioEdit (Hall 1999). The best-fitting nucleotide substitution model for the DNA matrix was determined in MEGA10 (Kumar et al. 2018) using maximum likelihood, comparing AICc and BIC. The selected model was GTR+I+G. The pairwise genetic distances were calculated in MEGA 12 under the Kimura 2-parameter model (K2P) using default settings. This choice followed a comparative criterion, as K2P has been used in the most comprehensive genetic studies of the genus (Costa Silva et al. 2015; Mejia and Buckup 2024a). The ultrametric tree was reconstructed under a Bayesian framework using BEAST 2.5 (Bouckaert et al. 2019), employing a Yule Process speciation model with a lognormal relaxed molecular clock. The nucleotide substitution model used was GTR. MCMC analyses were run for 10 million generations, sampling one tree every 10,000 generations. The first 25% of samples were discarded as burn-in, and the consensus tree was computed from the remaining 75%. Molecular Operational Taxonomic Units (mOTUs) were delimited using GMYC (multiple thresholds) (Fujisawa and Barraclough 2013) and bPTP (Zhang et al. 2013), both performed with default settings on the web server https://species.h-its.org/. Metadata for all sequences are provided in Suppl. material 1, and the aligned matrix is available in Suppl. material 2.

## Results

### Taxonomic account

#### Rineloricaria harenae sp. nov.

Taxon classificationAnimaliaSiluriformesLoricariidae

D2612F4B-DB3D-5145-8E6A-9C99E0D5535F

https://zoobank.org/34C61D92-7696-4D0B-8CF2-D4CFF0A215D7

Fig. 1, Table 1

Rineloricaria sp. 3—Costa e Silva et al. 2015 [molecular delimitation].

##### Type material.

***Holotype***: • LBP 35336, male (SL 92.1 mm); Brazil – Santa Catarina state • Jaraguá do Sul municipality, Itapocú River; 26°28'17.2"S, 49°10'55.1"W; R. Devidé, M.C. Chiachio, D.C. Ferreira & J.C.P. Alves leg.; 20 September 2005.

***Paratypes***: 19 specimens; same data as holotype; LBP 9162 (14), MZUSP 131542(5).

##### Diagnosis.

*Rineloricaria harenae* is distinguished from most of its congeners by having four lateral plate series in longitudinal rows below dorsal fin, mid-dorsal series not surpassing dorsal-fin spine [vs five lateral series, mid-dorsal series surpassing dorsal-fin spine in *R. aequalicuspis* Reis & Cardoso, 2001, *R. altipinnis* (Breder, 1925), *R. anhaguapitan* Ghazzi, 2008, *R. anitae* Ghazzi, 2008, *R. baliola* Rodriguez & Reis, 2008, *Rineloricaria buckupi* Mejia, Ferraro & Souto-Santo, 2025, *R. caracasensis* (Miranda Ribeiro, 1912), *R. cachivera* Urbano-Bonilla, Londoño-Burbano & Carvalho, 2023, *R. capitonia* Ghazzi, 2008, *R. caracasensis* (Bleeker, 1862), *R. daraha* Rapp Py-Daniel & Fichberg, 2008, *R. eigenmanni* (Pellegrin, 1908), *R. fallax* (Steindachner, 1915), *R. formosa* Isbrücker & Nijssen, 1979, *R. hasemani* Isbrücker & Nijssen, 1979, *R. heteroptera* Isbrücker & Nijssen, 1976, *R. isaaci* Rodriguez & Miquelarena, 2008, *R. jaraguensis* (Steindachner, 1909), *R. jubata* (Boulenger, 1902), *R. konopickyi* (Steindachner, 1879), *R. kronei* (Miranda Ribeiro, 1911), *R. latirostris* (Boulenger, 1900), *R. maacki* Ingenito, Ghazzi, Duboc & Abilhoa, 2008, *R. malabarbai* Rodriguez & Reis, 2008, *R. maquinensis* Reis & Cardoso, 2001, *R. melini* (Schindler, 1959), *R. microlepidogaster* (Regan, 1904), *R. morrowi* Fowler, 1940, *R. nudipectoris*, Mejia, Ferraro & Buckup, 2023, *R. osvaldoi* Fichberg & Chamon, 2008, *R. pentamaculata* Langeani & de Araujo, 1994, *R. phoxocephala* (Eigenmann & Eigenmann, 1889), *R. platyura* (Müller & Troschel, 1849), *R. quilombola*, Chamon & Fichberg, 2022, *R. reisi* Ghazzi, 2008, *R. rodriquezae* Costa-Silva, Oliveira & Silva, 2021, *R. rupestris* (Schultz, 1944), *R. steinbachi* (Regan 1906), *R. steindachneri* (Regan, 1904), *R. stewarti* (Eigenmann, 1909), *R. teffeana* (Steindachner, 1879), *R. tropeira* Ghazzi, 2008, *R. zaina* Ghazzi, 2008, and *R. zawadzkii* Costa-Silva, Silva & Oliveira, 2022]. It differs from remaining species of the genus, except for *R. setepovos* Ghazzi, 2008, by the absence of plates in the cleithral region [vs cleithral region fully or partially covered by plates in *R. atratoensis*Castellanos-Mejía et al. 2024, *R. aurata* (Knaack, 2003), *R. beni* (Pearson, 1924), *R. cadeae* (Hensel, 1868), *R. castroi* Isbrücker & Nijssen, 1984, *R. catamarcensis* (Berg, 1895), *R. cubataonis* (Steindachner, 1907), *R. felipponei* (Fowler, 1943), *R. giua* Castellanos-Mejía et al., 2024, *R. henselii* (Steindachner, 1907), *R. jurupari* Londoño-Burbano & Urbano-Bonilla, 2018, *R. lanceolata* (Günther, 1868), *R. langei* Ingenito, Ghazzi, Duboc & Abilhoa, 2008, *R. lima* (Kner, 1853), *R. longicauda* Reis, 1983, *R. magdalenae* (Steindachner, 1879), *R. microlepidota* (Steindachner, 1907), *R. misionera* Rodríguez & Miquelarena, 2005, *R. nigricauda* (Regan, 1904), *R. paraibensis* Mejia & Buckup, 2024, *R. pareiacantha* (Fowler, 1943), *R. parva* (Boulenger, 1895), *R. quadrensis* Reis, 1983, *R. sanga* Ghazzi, 2008, *R. sneiderni* (Fowler, 1944), *R. stellata* Ghazzi, 2008, *R. strigilata* (Hensel, 1868), *R. thrissoceps* (Fowler, 1943), *R. uracantha* (Kner, 1863), *R. wolfei* Fowler, 1940, *R. zawadzkii* Costa-Silva, Silva & Oliveira, 2022]. In having a group of plates in the central abdominal region connected to the pre-anal plates the new species differs from *R. setepovos*, which has the abdomen completely naked.

##### Description.

Morphometric data are provided in Table 1. Species small (maximum SL of analysed adults 92.1 mm). Dorsal profile of head slightly convex to straight from snout to posterior process of supraoccipital. Dorsal profile of body straight from supraoccipital to pectoral-fin insertion and remains slightly straight from this point to caudal-fin base. Dorsal margin of orbit elevated. Triangular to slightly elliptical snout with large naked area extending from snout tip to first pore of infraorbital branch of sensory canal. Eyes small (2.6–5.3% HL), dorsolaterally positioned with small postorbital notch, approximately ½ eye diameter. Lips covered with numerous small papillae randomly distributed. Lower lip exhibiting concentration of papillae around oral cavity. Lower lip larger than upper lip, not reaching pectoral girdle. Lip edge with discreet fringes and short ornamented maxillary barbel with reduced number of papillae. Both premaxilla and dentary bearing a row of slender, long, bicuspid teeth, with lateral cusp larger than medial cusp. Premaxilla with 6 or 7 (6) teeth and 6–8 (mode 6 or 7) teeth on dentary.

**Table 1.** Morphometric and meristic data of the holotype and 19 paratypes of *R. harenae* sp. nov. SD = standard deviation.

Measurement	Holotype	Min.	Max.	Mean	SD
Standard length (mm)	92.1	50.0	92.1	69.3	10.7
**% Standard length (SL)**					
Head length	19.9	14.1	21.3	19.5	0.6
Predorsal length	34.4	31.3	35.1	33.2	1.0
Postdorsal length	67.5	63.7	71.9	67.7	1.7
Prepectoral length	19.4	18.0	32.8	19.6	3.2
Postpectoral length	84.0	64.7	86.1	80.8	6.3
Prepelvic length	34.2	12.2	34.2	30.5	4.6
Postpelvic length	68.8	52.8	81.8	69.0	5.1
Preanal length	49.8	29.5	49.8	45.7	4.1
Postanal length	53.9	38.4	56.0	52.5	4.1
Unbranched dorsal-fin ray	19.7	18.6	21.3	20.1	0.7
Unbranched pectoral-fin ray	15.7	15.6	17.9	16.2	0.6
Unbranched pelvic-fin ray	14.0	14.0	16.3	14.8	0.8
Unbranched anal-fin ray	16.5	15.4	17.6	16.6	0.6
Superior unbranched caudal-fin ray	17.4	12.6	17.4	15.8	1.5
Inferior unbranched caudal-fin ray	N/A	10.3	13.3	12.4	0.8
Thoracic length	16.0	13.0	16.0	14.3	0.8
Abdominal length	18.1	15.1	18.1	16.8	0.7
Cleithral	19.7	15.3	19.7	17.2	1.1
Depth at dorsal-fin origin	9.3	8.1	11.2	9.3	0.9
Width at anal-fin origin	11.9	7.2	12.1	10.6	1.3
Caudal peduncle depth	1.4	1.2	1.6	1.4	0.1
Caudal peduncle width	3.4	1.4	3.6	3.1	0.5
**% Head length**					
Snout length	16.2	16.2	74.3	56.5	10.7
Eye diameter	15.6	8.8	29.0	16.7	4.1
Maximum orbital diameter	25.7	6.7	34.2	27.1	7.1
Interorbital width	24.2	4.9	32.6	24.8	7.1
Internarial width	7.9	5.4	10.3	7.5	1.0
Head depth	50.0	45.4	85.1	53.2	9.4
Head width	81.1	56.5	89.2	79.9	7.6
Free maxillary barbel	12.4	0.9	18.9	12.0	4.0
Ventro-rostral length	11.0	9.5	21.0	15.0	2.8
Lower lip	16.5	6.4	25.0	18.0	3.9

Ventral profile of head straight from snout tip to opercular region. Ventral profile of body slightly convex to caudal-fin base. Median lateral plates 26–28 (mode 27), incorporating laterosensory canals, with row of conspicuous keels. Anterior abdominal plates absent, leaving a large central area of pectoral girdle naked. Abdomen partially covered by plates. Abdominal plates of posterior complex start with polygonal pre-anal plate near urogenital pore. Pre-anal plate bordered anteriorly by three smaller plates, connected to small plates forming triangular group; these plates do not contact lateral abdominal plates and do not extend past pectoral-fin insertion. Rectangular lateral abdominal plates extend beyond pectoral-fin insertion.

Dorsal fin, I7 with its origin posterior to pelvic-fin insertion, with longest dorsal-fin ray extending beyond anal-fin origin. dorsal-fin spinelet present. Unbranched dorsal-fin ray slightly surpassing anal-fin origin. Pectoral-fin, I6, when positioned against body, reaches pelvic-fin insertion and, when extended, reaching anal-fin insertion. Pelvic-fin: i5, with its origin posterior to dorsal-fin insertion. Unbranched pelvic-fin ray convex and not extending to anal-fin origin. Anal-fin: I5, its origin posterior to posterior extremity of dorsal-fin rays. Adipose-fin absent. Caudal-fin: I10I, truncated, with upper unbranched caudal-fin ray prolonged into a short, thin filament.

##### Sexual dimorphism.

Sexually mature males exhibit hypertrophied odontodes, extending from the snout to the opercular region, as well as on the rays of the pectoral fin.

##### Colour in alcohol.

**Figure 1.**
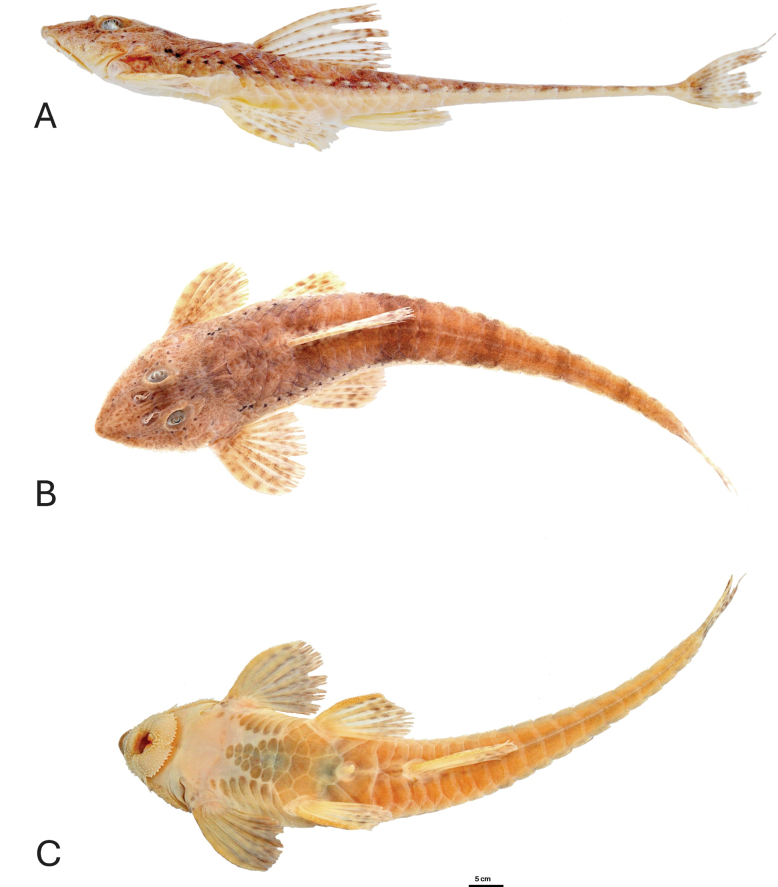
*Rineloricaria harenae*, male, LBP 35336, holotype, 92.1 mm SL, a specimen from Itapocú, municipitaly of Jaraguá do Sul, Santa Catarina, Brazil. **A**. Lateral view; **B**. Dorsal view; **C**. Ventral view.

Background colour of dorsal body surface light-brown (Fig. 1). Five transverse bars along dorsal surface, first at dorsal-fin insertion, second at end of dorsal-fin, and with three other transverse bars on caudal peduncle. Pores of laterosensory canal pigmented dark brown. Ventral region yellow-brown. Pectoral, pelvic, and dorsal fins with small horizontal stripes on rays. Anal fin hyaline with tiny irregular stripe, slightly darker near distal portion. Caudal fin with brown spot at base, stripes on central region and at tip.

##### Habitat and distribution.

**Figure 2.**
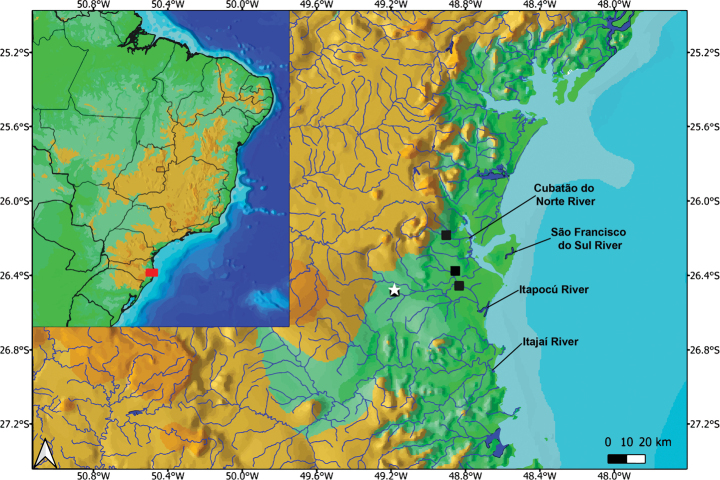
Distribution of *Rineloricaria harenae* and *R. jaraguensis* in the drainage basins of Santa Catarina, Brazil. Black squares indicate locations where *R. jaraguensis* samples were analysed. White star marks the type locality of *R. harenae* (26°28'17.2"S, 49°10'55.1"W).

*Rineloricaria harenae* was found exclusively at the type locality (Fig. 2), a second-order stream with moderate water velocity and a depth of < 1 m. The riparian vegetation at the type locality has been completely replaced by pasture, and early signs of siltation were observed, with sandbank accumulation where all studied specimens were collected. So far, the new species appears to have a restricted distribution, occurring only in a tributary of the Itapocú River in Santa Catarina, southern Brazil.

##### Etymology.

From harenae, the Latin word for sand, in reference to the sandy substrate characteristic of the habitat where the new species was discovered. Treated as a noun in apposition.

### Molecular results

**Figure 3.**
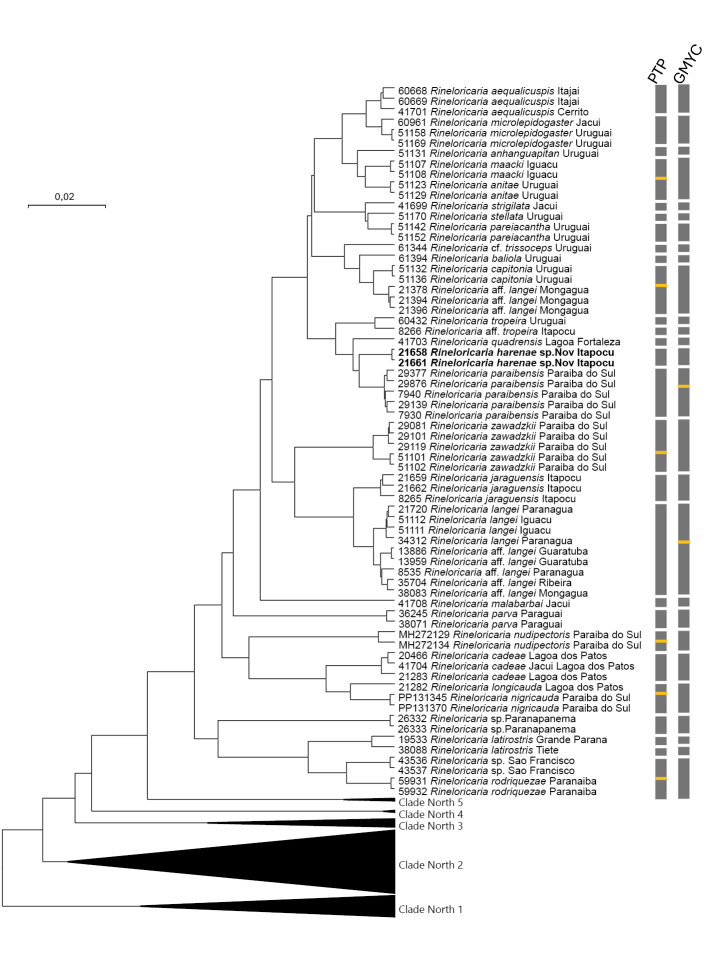
Bayesian phylogenetic tree of *Rineloricaria* species based on COI gene fragments. *Rineloricaria harenae* sp. nov. is highlighted in bold. Terminals are labelled with specimen voucher (or GenBank ID), species name, and sampled drainage, respectively. Bars on the right represent molecular Operational Taxonomic Units (mOTUs) inferred from bPTP and GMYC analyses; yellow divisions indicate incongruent delimitations between the two methods. Red asterisks mark nodes with posterior probability > 95%. The scale bar represents 2% genetic divergence under the K2P model. Amazonian clades are collapsed and shown as Clades North 1–5. See Suppl. materials 1, 2, 3, 4 for full details.

The final alignment of COI sequences, after editing, comprised 147 samples and 533 base pairs, of which 207 sites were variable, including eight singletons; no stop codons were detected. The phylogenetic analysis produced a tree with high statistical support at terminal nodes and lower support at basal nodes (Fig. 3). All morphologically recognized species were recovered as monophyletic. Both bPTP and GMYC analyses yielded largely congruent species delimitations: no analysis assigned more than one morphospecies to a single mOTU, although some morphospecies were split into multiple mOTUs, a pattern consistent with the results of Costa-Silva et al. (2015). Minor incongruences between bPTP and GMYC were observed concerning the delimitation of mOTUs within morphospecies.

The analyses successfully distinguished *R. harenae* from its closest congeners, *R. paraibensis* (2.3%) and *R. quadrensis* (2.7%), while divergence from other species ranged from 2.8% to 17.7% relative to the new species. Notably, genetic data positioned *R. harenae* in close phylogenetic proximity to a distinct group of species from the southern portion of the Eastern Basin (Fig. 3).

## Discussion

Integrative taxonomy has established itself as a powerful tool for the description of species within *Rineloricaria*. Since its pioneering application in this context just five years ago, 10 new species have been described from different basins in the Neotropical region (Costa-Silva et al. 2021; Mejia and Buckup 2024a; Mejia et al. 2023b; Castellanos-Mejía et al. 2024; Mejia et al. 2025c). Despite this progress, the genus still harbours considerable undescribed diversity, as previously indicated by Costa-Silva et al. (2015), whose study listed R. harenae as a candidate species (referred to as Rineloricaria sp. 3). Rineloricaria harenae is distinguished from its congeners by both molecular and morphological characteristics. bPTP and GMYC analyses confirm that it constitutes an independent evolutionary lineage (Fig. 3). The genetic distance from congeneric species exceeds the 2% threshold reported for Rineloricaria (Costa-Silva et al. 2015) and for other fish groups (Ward et al. 2009; Pereira et al. 2013; Shimabukuro-Dias et al. 2016). The most closely related congeners are R. paraibensis and *R. quadrensis*, from which the new species shows genetic divergences of 2.3% and 2.7%, respectively.

Morphologically, *R. harenae* can be easily distinguished from *R. paraibensis* and *R. quadrensis* by the presence of a naked area at the tip of the snout that extends posteriorly to the first pore of the laterosensory canal. In contrast, the latter two species possess an oval, non-elongated naked area. Additionally, *R. harenae* exhibits a unique combination of characters: ventral plate coverage is incomplete, featuring a naked scapular region and a centroabdominal area partially covered by a group of central plates, which connect only to the pre-anal plates (Fig. 1). All other characteristics follow the typical pattern of species in the sand group (sensu Rodriguez and Reis 2008).

Incomplete ventral coverage in *R. harenae* is an atypical trait for this group, usually associated with rock-dwelling species, which raises questions about its ecological function. Dermal plates are complex structures that provide effective protection against predators (Vickaryous and Sire 2009; Ebenstein et al. 2015; Lowe et al. 2021). However, ventral protection by plates appears to be a variably expressed trait among Loricariinae species (Londoño-Burbano and Reis 202 1; Dopazo et al. 2023), and even in some Rineloricaria species (Rodriguez and Reis 2008; Ghazzi 2008; Mejia et al. 2023b). Moreover, allometric variation in abdominal plate coverage during ontogenetic development has been documented in Loricariidae (Araujo and Langeani 2020). Moreover, allometric variation in abdominal plate coverage during ontogenetic development has been documented in Loricariidae (Araujo and Langeani 2020). Nevertheless, such variation was not observed in *R. harenae*, as all examined specimens—including juveniles—exhibited the same ventral coverage pattern.

Despite occurring in sympatry, *R. harenae* and *R. jaraguensis* are easily distinguishable by exhibiting the typical attributes of the sand group (except for ventral plating, as discussed) and the rock group (sensu Rodriguez and Reis 2008), respectively. They occupy distinct microhabitats within the same environment: *R. harenae* was collected exclusively from sandy banks, whereas *R. jaraguensis* was restricted to rocky substrates. This constitutes a classic case of sympatry without syntopy, i.e., geographical coexistence in the absence of ecological overlap.

### Comparative material

Collections studied: collection of Biologia e Genética de Peixes (LBP), Instituto de Biociências da Universidade Estadual de São Paulo; collection of Museu de Zoologia da Universidade de São Paulo (MZUSP).

*Rineloricaria aequalicuspis* Reis & Cardoso, 2001: LBP 14482, (32). *Rineloricaria anhaguapitan* Ghazzi, 2008: LBP 17032, (6). *Rineloricaria anitae* Ghazzi, 2008: LBP 11410, (2); 11416, (4). *Rineloricaria aurata* Knaack, 2003: LBP 681, (9); 9160, (1); 11176, (2); 11174, (5); 11175, (5). *Rineloricaria baliola* Rodriguez & Reis, 2008: LBP 14684, (3). *Rineloricaria cadeae* (Hensel, 1868): LBP 3336, (10); 8946, (1); 9159, (104); 11422, (15); 11432, (5); MZUSP 24578, (26). *Rineloricaria capitonia* Ghazzi, 2008: LBP 11414, (5); 11415, (19); 11418, (4). *Rineloricaria cubataonis* (Steindachner, 1907): LBP 2036, (32). *Rineloricaria fallax* (Steindachner, 1915): 11187, (7). *Rineloricaria jaraguensis* (Steindachner, 1909): LBP 2028, (9); 2363, (1); 3633, (25); 3644, (1); 728, (26); 730, (36). *Rineloricaria heteroptera* Isbrücker & Nijssen, 1976: LBP 1731, (4). *Rineloricaria kronei* (Miranda Ribeiro, 1911): LBP 771, (20); 7423, (21); MZUSP 058665, (5). *Rineloricaria lanceolata* (Günther, 1868): LBP 1557, (13); 2433, (3); 4367, (6); 8537, (6); 11173, (2); 802, (1); MZUSP 109865, (4). *Rineloricaria langei* Ingenito, Ghazzi, Duboc & Abilhoa, 2008: LBP 11401, (23). *Rineloricaria latirostris* (Boulenger, 1900): LBP 11266, (2); MZUSP 999, (1). *Rineloricaria longicauda* Reis, 1983: LBP 11430, (12). *Rineloricaria maacki* Ingenito, Ghazzi, Duboc & Abilhoa, 2008: LBP 11402, (1) (topotype); 11403, (6). *Rineloricaria malabarbai* Rodriguez & Reis, 2008: LBP 14455, (1). *Rineloricaria microlepidogaster* (Regan, 1904): LBP 14573, (15). *Rineloricaria nigricauda* (Regan, 1904): LBP 16845, (1); MZUSP 124042, (2). *Rineloricaria osvaldoi* Fichberg & Chamon, 2008: LBP 1650, (7). *Rineloricaria paraibensis*: LBP 6318, (12); LBP 6453, (2); MZUSP 058664, (5); 068353, (2); 090652, (4); LBP 802, (2); MZUSP 045455, (1). *Rineloricaria parva* (Boulenger, 1895): LBP 8471, (37). *Rineloricaria pentamaculata* Langeani & de Araujo, 1994: LBP 7357, (10). *Rineloricaria rodriquezae* Costa-Silva, Oliveira & Silva, 2021: LBP 11721, (7); 11741, (7). *Rineloricaria rupestris* (Schultz, 1944): LBP 6135, (1). *Rineloricaria stellata* Ghazzi, 2008: LBP 11419, (10). *Rineloricaria steindachneri* (Regan, 1904): (syntype photographed), NMW 45016; MZUSP 105202, (1). *Rineloricaria strigilata* (Hensel, 1868): LBP 3384, (24); 8941, (1). *Rineloricaria tropeira* Ghazzi, 2008: LBP 11404, (4); 11409, (1). *Rineloricaria zaina* Ghazzi, 2008: 11412, (5). *Rineloricaria* sp.: MZUSP 28995, (10); 28713, (8); 28697, (1); 24579, (10); 88334, (2); 83672, (6); 105266, (6).

## Supplementary Material

XML Treatment for Rineloricaria harenae

## Additional information

### Use of AI

No use of AI was reported.

### Author ORCIDs

Beatriz Rodrigues da Cruz https://orcid.org/0000-0003-1655-5310

Jonatas Santos Lima Pereira https://orcid.org/0000-0001-6175-8526

Claudio Oliveira https://orcid.org/0000-0002-7010-8880

Guilherme José da Costa Silva https://orcid.org/0000-0001-6380-8697

### Data availability

All of the data that support the findings of this study are available in the main text or Supplementary Information.

## Supplementary materials

Supplementary material 1 List of sequences used in the study Data type xlsx https://binary.pensoft.net/file/1540169This dataset is made available under the Open Database License (http://opendatacommons.org/licenses/odbl/1.0/). The Open Database License (ODbL) is a license agreement intended to allow users to freely share, modify, and use this Dataset while maintaining this same freedom for others, provided that the original source and author(s) are credited. Beatriz Rodrigues da Cruz, Jonatas Santos Lima Pereira, Claudio Oliveira, Guilherme José da Costa Silva

Supplementary material 2 Matrix used for molecular analysis Data type fas https://binary.pensoft.net/file/1540170This dataset is made available under the Open Database License (http://opendatacommons.org/licenses/odbl/1.0/). The Open Database License (ODbL) is a license agreement intended to allow users to freely share, modify, and use this Dataset while maintaining this same freedom for others, provided that the original source and author(s) are credited. Beatriz Rodrigues da Cruz, Jonatas Santos Lima Pereira, Claudio Oliveira, Guilherme José da Costa Silva

Supplementary material 3 Detailed bPTP results Data type pdf https://binary.pensoft.net/file/1540171This dataset is made available under the Open Database License (http://opendatacommons.org/licenses/odbl/1.0/). The Open Database License (ODbL) is a license agreement intended to allow users to freely share, modify, and use this Dataset while maintaining this same freedom for others, provided that the original source and author(s) are credited. Beatriz Rodrigues da Cruz, Jonatas Santos Lima Pereira, Claudio Oliveira, Guilherme José da Costa Silva

Supplementary material 4 Detailed GMYC results Data type png https://binary.pensoft.net/file/1540172This dataset is made available under the Open Database License (http://opendatacommons.org/licenses/odbl/1.0/). The Open Database License (ODbL) is a license agreement intended to allow users to freely share, modify, and use this Dataset while maintaining this same freedom for others, provided that the original source and author(s) are credited. Beatriz Rodrigues da Cruz, Jonatas Santos Lima Pereira, Claudio Oliveira, Guilherme José da Costa Silva
