# Supplementary material for: A new sand-dwelling species of Rineloricaria (Siluriformes, Loricariidae) from the Eastern Brazilian Basin
Source: Zookeys. 2026 Feb 13;1269:211–24. doi: 10.3897/zookeys.1269.155896 (PMC12924054; doi:10.3897/zookeys.1269.155896)
Supplement: Supplementary material 3 — Detailed bPTP results [file zookeys-1269-211_article-155896__-s003.pdf]

Maximum likelihood solution:

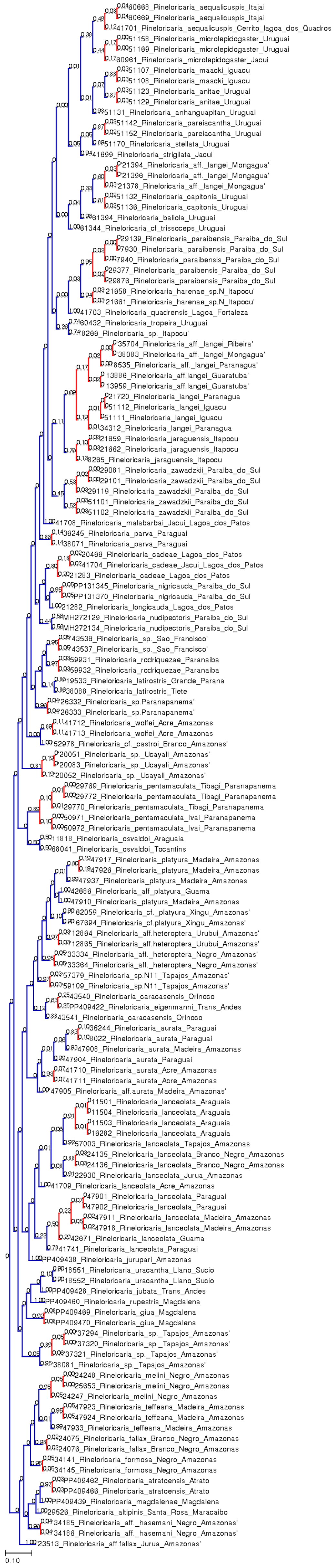

Highest Bayesian supported solution:

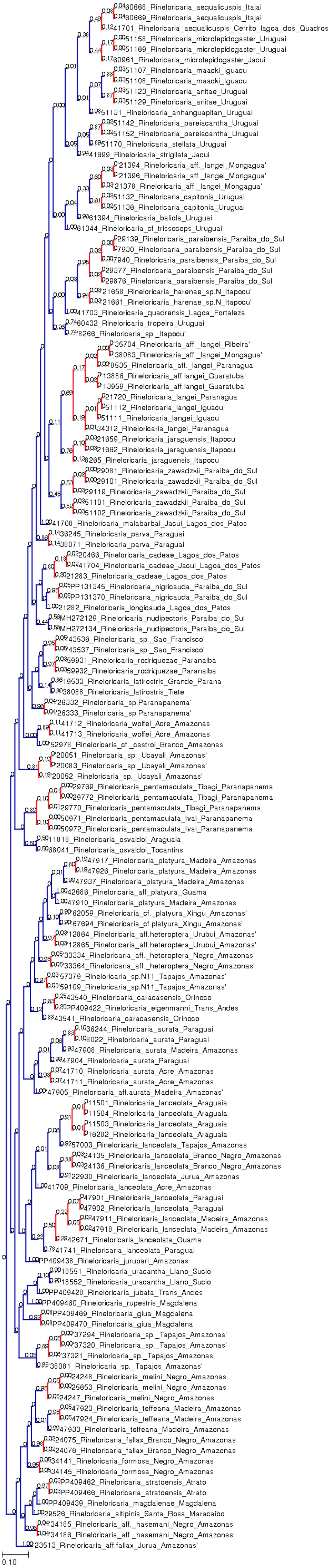

#Result of bPTP species delimitation
Accptance rate: 0.22392999999999999
Merge: 49744
Split: 50256
Estimated number of species is between 66 and 89
Mean: 79.52
