# Supplementary figures and images for: A new sand-dwelling species of Rineloricaria (Siluriformes, Loricariidae) from the Eastern Brazilian Basin
Source: Zookeys. 2026 Feb 13;1269:211–24. doi: 10.3897/zookeys.1269.155896 (PMC12924054; doi:10.3897/zookeys.1269.155896)

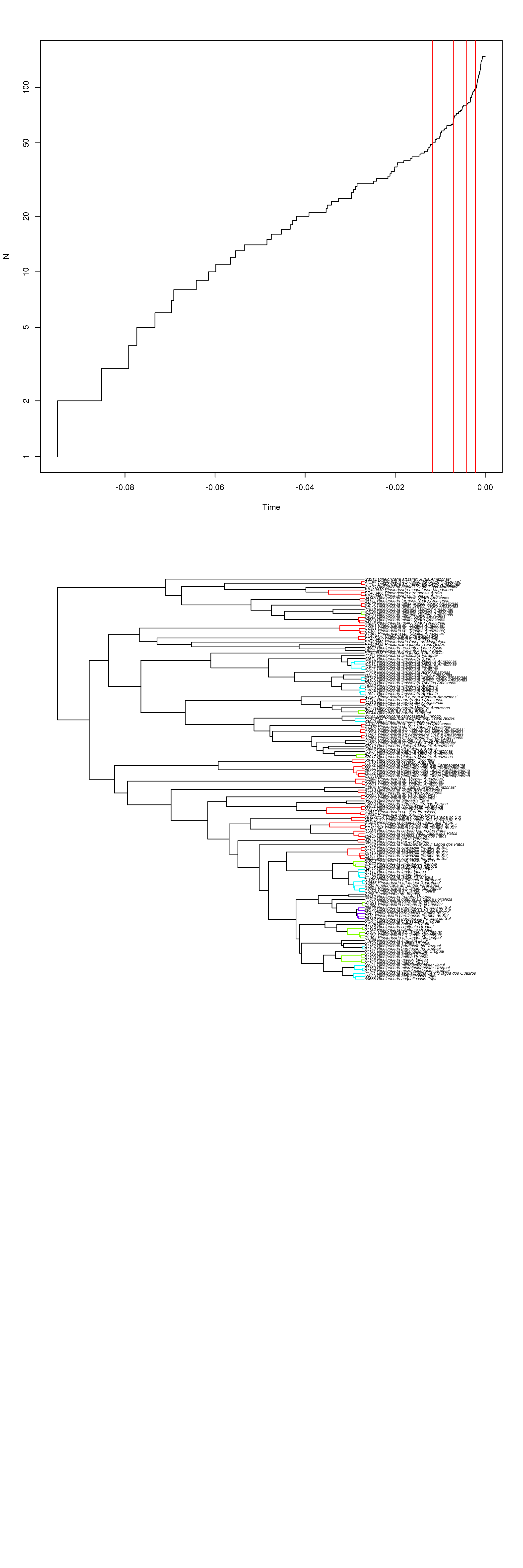

Supplement: Supplementary material 4 — Detailed GMYC results [file zookeys-1269-211_article-155896__-s004.png]
